# The cerebellum in dystonia: key player or background support?

**DOI:** 10.1055/s-0045-1814368

**Published:** 2025-12-22

**Authors:** Carlos Henrique Ferreira Camargo, Hélio Afonso Ghizoni Teive

**Affiliations:** 1 Universidade Federal do Paraná, Programa de Pós-Graduação em Medicina Interna, Disciplina de Doenças Neurodegenerativas, Curitiba PR, Brazil.; 2 Universidade Federal do Paraná, Departamento de Clínica Médica, Serviço de Neurologia, Curitiba PR, Brazil.

**Keywords:** Dystonia, Cerebellum, Purkinje Cells, Inferior Olivary Complex, Cholinergic Antagonists, Basal Ganglia

## Abstract

Since the 1960s, the pathophysiology of dystonia has been primarily attributed to dysfunction of the basal ganglia and their associated pathways. However, growing evidence from both basic and clinical research has highlighted the additional importance of the cerebellum, suggesting that dystonia arises from a motor-network dysfunction involving not only the basal ganglia, but also the cerebellum. Neuroimaging studies reinforce this concept, revealing structural and functional abnormalities in the cerebellum and its afferent pathways in patients with dystonia. Moreover, the dual involvement of the cerebellum and basal ganglia may help explain the frequent co-occurrence of dystonia in patients with ataxia and vice versa. The present review aims to integrate evidence from pathophysiology, clinical studies, genetics, and neuroimaging to underscore the crucial role of the cerebellum in the genesis of dystonia.

## INTRODUCTION

The term *dystonia* derives a combination of the Latin prefix *dis* - and the Greek suffix *tónos* . The word *tone* originally carried musical connotations. It stems from the 13th-century Old French *ton* (of the voice), from the Latin *tonus* —meaning *stretching* , *quality of sound* , *tone* , or *accen* t—which, in turn, also derives from the Greek suffix *tónos* , translated as *stretching* , *tension* , *raising of the voice* , or *pitch* . 1 According to the most recent consensus, dystonia is a movement disorder characterized by sustained or intermittent abnormal movements and/or postures. Dystonic movements and postures are typically patterned and repetitive, and they may appear tremulous or jerky. They are often triggered or exacerbated by voluntary actions, and they are frequently accompanied by overflow movements. 2

Since the 1960s, the pathophysiology of dystonia has been primarily associated with dysfunction of the basal ganglia and their pathways. 1 However, growing evidence from both basic and clinical research 3 4 has highlighted the additional importance of the cerebellum, suggesting that dystonia may result from a motor-network dysfunction involving not only the basal ganglia, but also the cerebellum.

The current narrative review aims to synthesize and organize the findings from various studies concerning the pathophysiology, genetics, clinical manifestations, neuroimaging, and treatment approaches that highlight the cerebellum's crucial role in the genesis of dystonia.

## METHODS

The current narrative review included original research articles, such as observational, cohort, cross-sectional, and case–control studies, as well as case series, clinical cases, meta-analyses, and reviews that addressed the interplay among genetics, clinical findings, neurophysiology, neuroimaging, and the cerebellar pathophysiology of dystonia.

A three-step search strategy was employed to ensure comprehensive coverage of the literature. First, a preliminary search was conducted in major databases (PubMed, Embase, and the Cumulative Index to Nursing and Allied Health Literature [CINAHL]) using the term *dystonia* combined with *ataxia* and *cerebellum* . This stage aimed to identify index terms, Medical Subject Headings (MeSH), and keywords from titles and abstracts of the retrieved papers. Second, a full search was performed using all identified keywords and index terms across the selected databases. Third, the reference lists of all retrieved studies were manually reviewed to identify additional relevant publications not captured by the electronic searches. Two reviewers (CHFC and HAGT) independently screened all titles and abstracts, resolving discrepancies through discussion. In addition, the references of the selected studies were carefully examined to identify further relevant sources.

Complementary to this strategy, the authors reviewed published articles and books on the history of dystonia, covering the earliest investigations into its pathophysiology and its proposed relationship with the cerebellum. The objective was to develop a narrative that critically examined the evolution of this relationship over time and introduced subsequent topics in a logical and coherent sequence.

## HISTORICAL ASPECTS

The term *dystonia* was first coined by Hermann Oppenheim (1858–1919) in his 1911 paper, 5 in which he described four unrelated children, all of Jewish origin, from Galicia (in Western Ukraine) and Russia. The standard features among these patients included twisted postures, intermittent sustained spasms, rapid and rhythmic movements, development of fixed postural deformities, and worsening during walking, all in the absence of weakness. Although Oppenheim initially considered hysteria or idiopathic bilateral athetosis as possible diagnoses, he ultimately recognized this as a new hereditary disorder and proposed the name *dystonia musculorum deformans* . 5

As Oppenheim initially hypothesized with hysteria, dystonia often lingered under an ambiguous definition between a neurological and a psychiatric disorder. At times, it was even proposed that dystonia should not be considered a distinct movement disorder. 1 Jean-Martin Charcot (1825–1893), known as the “Father of Neurology,” showed a great interest in the study of hysteria and frequently presented patients with movement abnormalities that would today be classified as functional movement disorders, including functional dystonias. 6 In his 1908 doctoral thesis, 7 Marcus Walter Schwalbe (1883-1926) had already recognized dystonia as a condition distinct from previously-described movement disorders. He also identified its hereditary nature, which he observed in a Jewish family. Nevertheless, Schwalbe considered the disorder to be partly psychiatric, referring to it as a “tonic cramps syndrome with hysterical symptoms.” 7

Oppenheim also considered the possibility of diagnosing athetosis. 5 The term *athetosis* , from the Greek meaning “without fixed position,” had been coined earlier, in 1871, by William Alexander Hammond (1828–1900), author of the first American neurology textbook. Although Hammond struggled to establish athetosis as a distinct clinicopathological entity—and despite his accurate prediction of striatal pathology in his initial case—many neurologists regarded athetosis as merely a form of post-hemiplegic chorea or as part of a chorea–dystonia continuum. 8 9 Nevertheless, his pathological observations encouraged contemporary physicians and researchers to associate such movement disorders with irritation or damage to the basal ganglia and thalamus, rather than with pyramidal tract dysfunction. 9

Despite this early understanding of the role of the basal ganglia in movement disorders—and the vigorous debate nearly 4 decades later on the very nosographic status of dystonia at the Tenth International Neurological Meeting in Paris, in 1929, with contributions from Ludo van Bogaert (1897–1989), Jules Froment (1878–1946), Gheorghe Marinescu (1863–1938), Jean Alexandre Barré (1880–1967), Henry Meige (1866–1940), and Jean Lhermitte (1877–1959)—neurological interest in dystonia subsequently waned for more than a decade. During this period, psychiatrists, strongly influenced by psychoanalytic thought, advanced Freudian explanations for the contorted and disfiguring postures observed in the focal and generalized forms of dystonia. 1 9

This situation began to change between the 1940s and 1970s, when clinicians such as Ernest Herz (1901–1966) and Wolfgang Zeman (1921–2001) revisited the phenomenology of dystonia, emphasizing its neurological basis. Their detailed clinical observations highlighted the organic nature of the disorder and paved the way for a renewed interest in its classification. 10 11 During the same period, Derek Denny-Brown (1901–1981) published 3 classic monographs: *Diseases of the Basal Ganglia and Subthalamic Nuclei* (1946), *The Basal Ganglia* (1962), and *The Cerebral Control of Movement* (1966). These works provided, for the first time, an in-depth discussion of the anatomy and physiology of the basal ganglia in relation to several movement disorders. In his most influential volume, *The Basal Ganglia and Their Relation to Disorders of Movement* , he also examined classic diseases such as dystonias. 12 The true revival of interest, however, occurred in the 1970s, driven mainly by David Marsden (1938–1998), who redefined the phenomenology, emphasized the variability in clinical presentations, and reframed dystonia as a primary movement disorder caused by dysfunction of the basal ganglia. 13

In 1886, William Gowers (1845–1915) reported that writer's cramp could sometimes be preceded by trauma or local disease. 14 According to the current understanding, following a peripheral injury, any alteration in normal anatomy and physiology may result in peripherally-induced movement disorders (PIMDs), which also include certain task-specific disorders arising after repetitive activities, often referred to as *overuse syndromes* . Some examples are musicians' dystonia, sports-related dystonia, and other movement disorders in performing artists, which often develop after years of highly-skilled, precise motor practice, or following a recent increase in training intensity. 15

**Figure 1:**
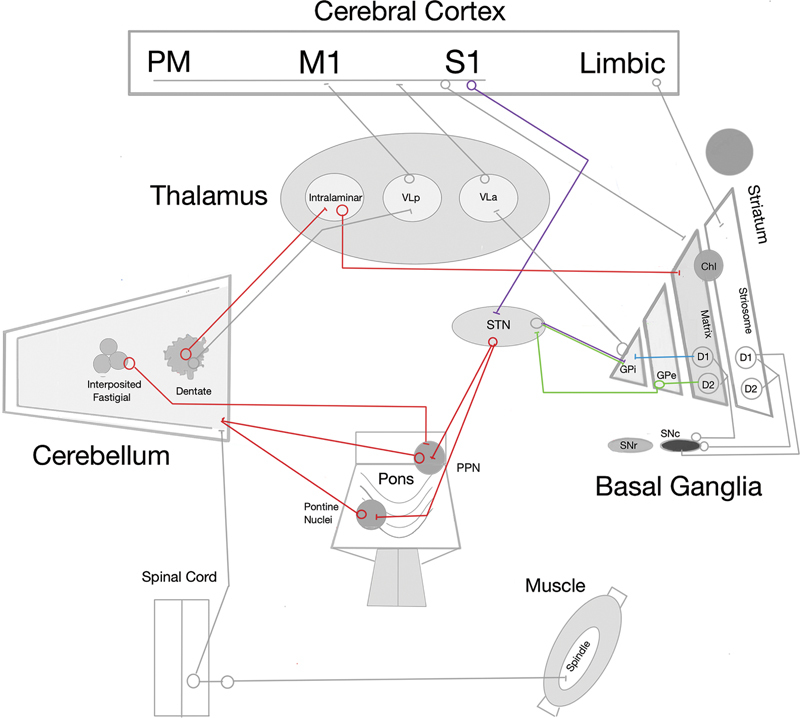
Abbreviations: ChI, cholinergic interneuron; D1 and D2, dopaminergic neurons; GPi, , globus pallidus internus; GPe, globus pallidus externus; PPN, pedunculopontine nucleus; SNc, substantia nigra pars compacta; SNr, substantia nigra pars reticulata; STN, subthalamic nucleus; VLa and VLp, ventral lateral anterior nucleus; VLp, ventral posterior nucleus. Cerebral cortex: M1, primary motor area; PM, premotor area; S1, primary somatosensory area. Simplified diagrams illustrating the main interrelationships among brain structures associated with dystonia. Classic cortical-basal ganglia-thalamocortical pathways: direct pathway (blue), indirect pathway (green), hyperdirect pathway (purple). Main cerebellar connections (red). Adapted from Kaji, 19 Camargo et al., 20 and Wang et al. 21

Peripheral lesions may contribute to the generation of dystonia through pathways involving the cerebellum. 16 17 In 1924, Francis Martin Walshe (1885–1973) reported 18 that a diluted local anesthetic could improve akinesia in a patient with Parkinson's disease, reasoning that this effect was mediated by alterations in muscle afferent activity. Decades later, in 1995, Ryuji Kaji (1935–) injected diluted lidocaine into dystonic muscles and demonstrated that temporary blockade of muscle afferents could transiently suppress abnormal contractions. 17 When injected diffusely into a muscle, local anesthetics predominantly affect gamma efferent fibers that innervate muscle spindles. Because muscle afferents constitute the major input for kinesthetic sensation, and spindle afferent pathways project densely to the cerebellum via the spinocerebellar tracts, these findings underscore the importance of altered sensory feedback in motor control ( Figure 1 ). 16 19 20 21 Neuroimaging studies further support this concept, revealing structural and functional abnormalities in the cerebellum and its afferent pathways in patients with dystonia. 3 Taken together, these observations support the current hypothesis that dystonic movements and postures may be preprogrammed within subcortical circuits, even at rest, reflecting abnormal sensorimotor integration. Nowadays, although these pathways remain only partially understood, substantial anatomical evidence confirms the existence of direct disynaptic connections between the cerebellum and both the striatum and the subthalamic nucleus (STN, Figure 1 ). 16

## PATHOPHYSIOLOGY

### Basal ganglia

Since the 1990s, the predominant clinical model of movement disorders involving the basal ganglia has proposed the existence of two primary opposing neural circuits, the direct and indirect pathways (commonly referred to as the *Go* and *No-Go* pathways). 22 According to this framework, balanced activity between these circuits is essential for normal motor control, whereas their imbalance results in either hypo- or hyperkinetic movement disorders. Nevertheless, controversies regarding the direct–indirect pathway model have steadily accumulated, and this framework has never adequately explained the pathophysiological origins of dystonia. 23

According to the dual Go/No-Go model, D1 receptor-expressing striatal projection neurons (D1SPNs) that project to the globus pallidus internus (GPi) initiate the pallido-thalamocortical system (and the pallido-nigro-thalamocortical system), forming the direct pathway that facilitates movement. In contrast, those expressing D2 receptors (D2SPNs) to the globus pallidus externus (GPe), which projects to the STN, which, in turn, projects to the GPi, form the indirect pathway, which inhibits movement ( Figure 1 ). 22 23 Over the past 4 decades, however, this framework has undergone extensive revisions. Experimental studies 23 have shown that D1SPNs and D2SPNs can be simultaneously active and cooperate, suggesting roles in reinforcement-based learning rather than a strict Go/No-Go dichotomy.

In fully-learned motor tasks, the pathway affected in dystonia is classified as a reinforcement learning circuit within the basal ganglia mediated by the dopaminergic system. As motor skills and efficiency improve, there is a shift from supervised to reinforcement learning, with greater involvement of the basal ganglia. 19 24 However, if excessive dopamine was the sole cause of dystonia, important clinical conditions would remain unexplained, such as tardive dystonia and dopa-responsive dystonia. Although atypical antipsychotic drugs are associated with a lower risk of tardive syndromes, a substantial number of psychiatric patients continue to develop dystonia while using dopaminergic antagonists. Conversely, patients with dopa-responsive dystonia show dramatic improvement with levodopa treatment. 19

Therefore, reinforcement learning models have further reshaped the interpretation of basal ganglia function, demonstrating that stimulation of D1SPNs is reinforcing and stimulation of D2SPNs is aversive, with pathway roles shown to be conditional and experience-dependent. Anatomical discoveries have added additional complexity: the hyperdirect pathway provides a rapid bypass of striatal processing, and the GPe has emerged not as a passive relay but as a key regulatory hub that dynamically shapes basal ganglia output through its projections to the STN, GPi, and striatum ( Figure 1 ). Cell-type–specific studies have shown that distinct GPe neuronal populations exert opposing influences on these downstream targets, enabling the GPe to fine-tune movement rather than merely transmit signals. This expanded view underscores the limitations of the classical model and reinforces the need for a more dynamic framework of basal ganglia circuitry 21 .

A recent study 23 has revealed that the canonical direct–indirect model is echoed by a parallel organization arising from the striosome compartment of the striatum. Unlike the matrix-derived pathways, which project to motor-output nuclei, the striosomal direct (S-D1) and indirect (S-D2) pathways target dopamine-containing neurons of the substantia nigra pars compacta (SNc). The striosome compartment receives prominent inputs from limbic-related cortical regions, linking these circuits to motivation, mood, and decision-making ( Figure 1 ). Remarkably, the striosomal pathways exert effects opposite to those of the canonical pathways: activation of S-D1 reduces movement and dopamine release, while activation of S-D2 increases movement. This parallel organization suggests that striosomes provide homeostatic modulation of basal ganglia output and may contribute to non-motor symptoms. 23 Remarkable histopathological loss of the striosome compartment in the striatum is found in the dystonic phase of X-linked dystonia-parkinsonism (XDP). 25 26

Thus, while the direct–indirect pathway model remains a valid starting point, its initial simplicity has proven insufficient to explain complex movement disorders such as dystonia. Contemporary understanding recognizes that basal ganglia circuits operate through dynamic cooperation between pathways, integration with reinforcement learning, and conditional and context-dependent functions, as well as critical contributions from structures such as the GPe and the hyperdirect pathway. These revisions underscore the strengths and limitations of the canonical model, highlighting the necessity of moving beyond the traditional Go/No-Go framework. 23

### Cerebellum and its pathways

Traditionally, the basal ganglia and the cerebellum were considered independent motor circuits that only communicated indirectly through cortical relays. However, anatomical and physiological evidence 16 have demonstrated the existence of direct disynaptic connections between these structures. Specifically, the dentate nucleus projects to the striatum via the intralaminar nuclei of the thalamus, while the STN provides disynaptic outputs to the cerebellar cortex through the pontine nuclei or pedunculopontine nucleus ( Figure 1 ). 4 19 27 28 29 Electrophysiological studies 30 in awake mice have confirmed that these pathways enable the cerebellum to modulate striatal activity with short latency (∼ 10 ms).

In the intact system, cortical high-frequency stimulation typically induces long-term depression (LTD) at corticostriatal synapses. When cerebellar input is coactivated, however, synaptic plasticity shifts towards long-term potentiation (LTP). 30 This suggests that the cerebellum plays a crucial role in shaping corticostriatal plasticity, thereby influencing the integration of sensory, motor, and dopaminergic signals necessary for fine-tuned motor control. 19 Cholinergic interneurons (ChIs)—strategically positioned at the border between the striosome and matrix striatum compartments—appear to be key mediators of this regulation, as they modulate dopamine release through nicotinic and muscarinic receptors. 31 32 In dystonia, this physiological balance is disrupted by aberrant cerebellar inputs. 19 Experimental models have shown 30 that abnormally high-frequency cerebellar signals bias corticostriatal synapses toward pathological LTP, impairing the ability to depotentiate. This maladaptive plasticity has been demonstrated in models of dopa-induced dyskinesia 33 and in patients with *DYT1* ( *TOR1A* ) dystonia, 34 35 suggesting a shared mechanism of abnormal synaptic reinforcement.

A recent study 36 using genetic mouse models and electrophysiological recordings has provided new clarity, particularly through the identification of disease-specific spike signatures in the interposed cerebellar nucleus. In carefully designed models, disruption of Purkinje cell Gamma-aminobutyric acid (GABA)-ergic transmission in *Pcp2 ^Cre^* and *Slc32a1 ^fl/fl^* mice reliably produced ataxia. By contrast, the selective elimination of glutamatergic climbing fiber input to Purkinje cells in *Ptf1a ^Cre^* and *Slc17a6 ^fl/fl^* mice resulted in twisting postures and limb hyperextension, consistent with dystonia. 36

Recordings from the interposed nuclei have revealed that each disorder was associated with distinct neuronal firing patterns, or spike signatures. 36 Ataxia was associated with two distinct yet consistent signatures, whereas dystonia exhibited its own stereotyped patterns. Notably, the severity of dystonia correlated with the balance between normal and pathological activity: mice with mild, stress-triggered dystonia retained more neurons exhibiting control-like firing, whereas severe models, such as ouabain-infused or *Ptf1a ^Cre^* and *Slc17a6 ^fl/fl^* mice, showed a predominance of neurons with a dystonia-specific signature. These findings establish a direct link between the relative proportion of neurons expressing pathological activity in the interposed nucleus and the clinical severity of dystonia. Perhaps most compelling, optogenetic induction of these spike signatures in otherwise normal mice was sufficient to reproduce the characteristic motor behaviors of ataxia or dystonia. This demonstrates that the pattern of cerebellar output, rather than structural lesions, is the critical determinant of phenotype. 36

While most attention has historically focused on cerebellar efferents from the dentate nucleus, these findings underscore the pivotal role of other cerebellar nuclei. Through its extensive connections with the pedunculopontine nuclei, reticular formation, and other brainstem centers, the interposed nucleus emerges as a central hub through which cerebellar dysfunction may diversify into distinct motor syndromes.

## CLINICAL AND GENETIC INSIGHTS

Many genetic diseases display complex phenotypes resulting from neurodegeneration across multiple systems and structures, including the basal ganglia and the cerebellum. This dual involvement may help explain the frequent co-occurrence of dystonia in patients with ataxia and other neurological manifestations. Network-based approaches have also provided compelling evidence 3 4 37 38 that the molecular pathways underlying ataxia and dystonia are closely interconnected.

Cerebellar ataxia and dystonia have distinct physiological correlates; yet, they share specific abnormalities in muscle activation patterns, including prolonged agonist activity and mistimed recruitment of antagonists. Cerebellar ataxia may arise from two different mechanisms: loss of cerebellar function or, conversely, an abnormal gain in function. In patients with ataxia attributed to loss of function, the pattern of agonist–antagonist activation differs from that observed in dystonia. However, in a subset of ataxic patients, the activation pattern overlaps with that of dystonia, suggesting that their ataxia may reflect an abnormal gain in cerebellar output. Such gain-in-function phenomena may also occur occasionally in the spinocerebellar ataxias, in which ataxia and dystonia coexist, and the most pronounced morphological changes are found in the cerebellum. Viewed in this way, progressively-increasing abnormal gain in cerebellar function may represent a continuum that includes dystonia, in which cerebellar output fails to adequately distinguish signals intended for agonist and antagonist muscles, thereby producing co-contraction and motor overflow. 39

A critical role for the cerebellum in the pathophysiology of dystonia 4 37 is also supported by multiple lines of clinical evidence, including lesion location in secondary dystonia, 40 41 the syndrome of dystonia with cerebellar atrophy (DYTCA), 42 and postmortem pathological findings in cervical dystonia. 43 Moreover, dystonia may be a presenting or prominent feature in several autosomal dominant hereditary ataxias; however, it is essential to note that these are multisystemic diseases characterized by degeneration that is not confined to the cerebellum ( Table 1 ). 4 44 45 46 47 48 49 50 51 52 53 54 55 56 57

**Table 1:** Prevalence and clinical features of dystonia across SCAs

SCA	Prevalence of dystonia	Clinical features	References
SCA- *ATXN1* SCA1	12–13%	Associated with a greater severity of ataxia	44 45
SCA- *ATXN2* SCA2	14–18%	Associated with a greater severity of ataxia	44 45
SCA- *ATXN3* SCA3-MJD	∼ 24%	Associated with a greater severity of ataxia	44 45
SCA- *CACNA1A* SCA6	5–9%	Slower progression; mainly cerebellar cortex involvement	44 45
SCA- *ATXN7* SCA7	Case reports	Craniocervical dystonia; writer's cramp; cerebellar and brainstem atrophies	46 47
SCA- *ATXN8OS* SCA8	Case report	Oromandibular dystonia; cerebellar atrophy	48
SCA- *PPP2R2B* SCA12	Case report	Cervical dystonia; dysphonia; tremor	49
SCA- *PRKCG* SCA14	∼ 32%	Focal/task-specific dystonia; myoclonus is frequent	50
SCA- *TBP* SCA17	Frequent	Chorea; multiple dystonias (blepharospasm, torticollis, writer's cramp, foot dystonia)	51
SCA- *KCND3* SCA19/22	Rare cases	Childhood onset with ataxia, dystonia, myoclonus, and cognitive impairment	52
SCA- *TGM6* SCA35	∼ 12%	Cranial dystonia; head tremor with DBS response	53 54
SCA- *NOP56* SCA36	Case reports	Focal dystonia; dystonic tremor; late onset	55
SCA- *STUB1* SCA48	Case reports	Cognitive decline; focal dystonia; chorea; myoclonus	56
SCA- *ATN1* DRPLA	Case reports	Focal/segmental dystonia; often with chorea and myoclonus	57

Abbreviations: CBS, deep brain stimulation; DRPLA, dentatorubral-pallidoluysian atrophy; MJD, Machado-Joseph disease; SCA, spinocerebellar ataxia.

Autosomal recessive cerebellar ataxias (ARCAs) also constitute a heterogeneous group in which dystonia may occur as part of the clinical phenotype. The most prevalent ARCAs associated with dystonia include Friedreich ataxia ( *FXN* ), ataxia-telangiectasia ( *ATM* ), ataxia with isolated vitamin E deficiency ( *TTPA* ), *POLG* -related disorders, ataxia with oculomotor apraxia types 1, 2, and 4 ( *APTX* , *SETX* , and *PNKP* respectively), *PRRT2* -associated disorders, and autosomal recessive spinocerebellar ataxia type 5 or Galloway–Mowat syndrome ( *WDR73* ). 3

When dystonia is the only presenting feature aside from a possible tremor, it is referred to as *isolated dystonia* . In contrast, dystonia associated with another movement disorder or as part of broader neurological or systemic syndromes is classified as *combined dystonia* . The first isolated dystonia gene, *TOR1A* , was identified almost 30 years ago. Since then, the number of known genetic forms has increased significantly, primarily due to the advancement in next-generation sequencing (NGS) technologies. 2 Importantly, some monogenic dystonias, both isolated and combined, may occasionally manifest with ataxia ( Table 2 ). 58 59 60 61 62 63

**Table 2:** Phenotypic spectrum of dystonia-related genes ( *DYT* ) with ataxia overlap

Dystonia	Phenotypic spectrum and clinical features	References
DYT- *TUBB4A* DYT4	*TUBB4A* encompasses a broad spectrum, ranging from DYT4 to hypomyelination with atrophy of the basal ganglia and cerebellum (H-ABC) leukoencephalopathy, characterized by symptoms including dystonia, psychomotor delay, spasticity, ataxia, dysarthria, short stature, and microcephaly. DYT4 should not be categorized as an isolated dystonia.	58
DYT- *THAP1* DYT6	Clinically heterogeneous. A reported case demonstrated dystonia followed by the subsequent development of ataxia; transcranial magnetic stimulation (TMS) revealed absent cerebellar inhibition (CBI), supporting cerebellar involvement in the phenotype.	59
MYC/DYT- *SGCE* DYT11	Classically, myoclonus-dystonia with childhood onset, but atypical presentations may include episodic ataxia and opsoclonus-myoclonus-like features.	60
DYT/PARK- *ATP1A3* DYT12	*ATP1A3* is associated with a broad neurological spectrum, including alternating hemiplegia of childhood, rapid-onset dystonia-parkinsonism, cerebellar ataxia-areflexia-pes cavus-optic atrophy-sensorineural hearing loss (CAPOS) syndrome, relapsing encephalopathy with cerebellar ataxia (RECA), and even psychiatric syndromes.	61
DYT- *ANO3* DYT24	Slowly-progressive dystonia with facial grimacing, eyelid spasms, unstable gait, and progressive dysarthria/ataxia. One reported patient had prior midbrain and cerebellar infarction, but dystonia/ataxia progressed independently.	62
DYT- *KMT2B* DYT28	Novel splice-site variant described in a patient with adult-onset ataxia, mild dystonia, neuropathy, seizures, and ophthalmological involvement. Highlights the potential role of *KMT2B* in hereditary ataxias beyond dystonia.	63

Neuroimaging studies 64 in *DYT1* ( *TOR1A* ) support the view that dystonia is a network and neurodevelopmental disorder, highlighting the role of imaging in elucidating its pathophysiology. Carriers of the *TOR1A* guanine-adenine-guanine (GAG) deletion exhibit metabolic changes in specific brain regions, characterized by reduced GABA and D2 receptor availability in the striatum, as well as abnormalities in the cerebello-thalamocortical pathway. This circuit can be divided into proximal (cerebellothalamic) and distal (thalamocortical) components: abnormalities restricted to the proximal part have been demonstrated in manifesting carriers. They are linked to increased activation of the supplementary motor area (SMA). In contrast, combined proximal and distal abnormalities occur in non-manifesting carriers, thought to prevent the spread of abnormal impulses to the cortex and thereby protect against clinical dystonia. In addition, normal fractional anisotropy in the superior cerebellar peduncle has been observed 64 in a patient with the 216H polymorphism, a finding associated with preservation of normal cerebello-thalamocortical connectivity.

## TREATMENT INSIGHTS

### Pharmacological treatments

The traditional understanding has been that anticholinergics alleviate dystonia by correcting a striatal neurotransmitter imbalance, characterized by reduced dopaminergic and increased cholinergic activities. 65 However, studies 66 67 have expanded this view, suggesting that their beneficial effects may also involve the modulation of cerebellar circuits.

Within the striatal matrix, giant aspiny interneurons—also known as *ChIs* —serve as an intrinsic source of acetylcholine (Ach), whereas the pedunculopontine nucleus provides extrinsic cholinergic input. 68 Cholinergic interneurons are strongly modulated by thalamostriatal innervation, particularly from the cerebellar dentate nucleus via the intralaminar thalamus, more so than by corticostriatal input. Repetitive thalamic stimulation increases ChI firing, linking cerebellar output to striatal cholinergic activity. Dysfunction of ChIs is thought to contribute to abnormal motor pattern selection, consistent with both striosomal and cerebellar hypotheses of dystonia pathogenesis. Significantly, ChI activation can trigger dopamine release by engaging presynaptic nicotinic receptors, while striosomal dysfunction facilitates additional dopamine release through striosome–SNc circuits ( Figure 1 ). Together, these processes converge to produce dopamine excess and increased movement amplitude via facilitation of the direct pathway. 23 29

Cerebellar neurons dynamically adjust their firing frequency according to the activation of agonist or antagonist muscles during motor tasks, with distinct Purkinje cells and downstream nuclear neurons likely involved in the precise timing of this activity. 39 Acetylcholine also plays a critical role in cerebellar function, with dense cholinergic projections terminating in the granule cell layer, thereby exerting a significant influence on cerebellar processing and associated behaviors. 69 Fore et al. 69 demonstrated in vitro that ACh produces a prolonged inhibitory effect on Golgi cells via muscarinic receptor activation, thereby reducing the inhibitory drive onto granule cells. Simultaneously, muscarinic receptor activation on mossy fibers diminishes excitatory input to granule cells. This concurrent reduction in excitation and inhibition alters spike probability in a heterogeneous manner—enhancing excitability in some granule cells while suppressing it in others. Significantly, ACh preferentially increases the excitability of strongly-inhibited granule cells, supporting the concept that granule cell inhibition is stimulus-specific and essential for cerebellar learning. These findings suggest that cholinergic neuromodulation can selectively enhance learning for specific mossy fiber inputs depending on behavioral context or stimulus salience. 69 Thus, ACh may serve as a key regulator of cerebellar plasticity, modulating the gain in signal processing from granule cells through Purkinje cells to the deep cerebellar nuclei. 39 Nevertheless, the in-vivo mechanisms that govern cerebellar ACh release and its precise impact on synaptic and network dynamics remain incompletely understood. 69

### Neurosurgical treatment

Deep brain stimulation (DBS) has been applied to different targets in dystonia, including the GPi, the ventrointermediate nucleus (VIM), and the STN. 19 70 The GPi remains the most established target, and it is generally considered the first choice. 19 70 However, GPi-DBS and STN-DBS are safe and effective, producing substantial improvements in patients' quality of life. 70 However, the choice of the optimal target remains the subject of debate. 70

The rationale for GPi-DBS is better established: by increasing GABAergic inhibition from the GPi to the ventrolateral thalamus, it reduces thalamocortical excitatory drive to the premotor cortex, which is characteristically hyperexcitable in dystonia ( Figure 1 ). In contrast, the mechanisms underlying STN-DBS remain less clearly defined. Some authors 19 39 have suggested that dystonia may involve an imbalance with relative predominance of the direct pathway, and that STN stimulation could restore basal ganglia output by engaging the indirect pathway. However, this interpretation is uncertain. Alternative hypotheses propose that STN-DBS may modulate sensorimotor integration through the activation of either orthodromic thalamocortical or antidromic hyperdirect pathways. A particularly compelling possibility is that the delayed clinical improvement often observed after STN-DBS reflects adaptive changes in disynaptic projections from the STN to the cerebellar cortex via the pontine nuclei or pedunculopontine nucleus, thereby influencing cerebello-thalamocortical circuits ( Figure 1 ). This perspective emphasizes the broader network interactions underlying dystonia. 19 39

## CONCLUSION

Although the pathophysiology of dystonia has not been fully elucidated yet, it appears to be complex and involves multiple brain structures and their interconnected pathways. After being dismissed for decades as purely psychogenic, dystonic movements were subsequently attributed to imbalances within basal ganglia circuits. However, with the accumulation of evidence from recent studies and advances in neuromodulation techniques, such as DBS, it has become evident that these mechanisms are far more intricate. Today, it is clear that the cerebellum, in conjunction with the basal ganglia, plays a crucial role in the development of dystonia. Future investigations will likely clarify these contributions in greater detail, as well as uncover the involvement of additional structures currently regarded as secondary players. A deeper understanding of these circuits and their neurotransmitters will be crucial for the development of novel therapeutic strategies.
